# Multi-use physical activity trails in an urban setting and cardiovascular disease: a difference-in-differences analysis of a natural experiment in Winnipeg, Manitoba, Canada

**DOI:** 10.1186/s12966-022-01279-z

**Published:** 2022-03-28

**Authors:** Jonathan McGavock, Erin Hobin, Heather J. Prior, Anders Swanson, Brendan T. Smith, Gillian L. Booth, Kelly Russell, Laura Rosella, Wanrudee Isaranuwatchai, Stephanie Whitehouse, Nicole Brunton, Charles Burchill

**Affiliations:** 1 Department of Pediatrics and Child Health, Rady Faculty of Health Sciences, University of Manitoba, Winnipeg, Canada; 2 Diabetes Research Envisioned and Accomplished in Manitoba (DREAM) Theme, Children’s Hospital Research Institute of Manitoba, 715 McDermot Ave, Winnipeg, MB R3E 3P4 Canada; 3 Public Health Ontario, Toronto, Canada; 4 Manitoba Centre for Health Policy, Rady Faculty of Health Sciences, University of Manitoba, Winnipeg, Canada; 5 Winnipeg Trails Association, Winnipeg, Canada; 6 Dalla Lana School of Public Health, University of Toronto, Toronto, Canada; 7 MAP Centre for Urban Health Solutions, St. Michaels Hospital, Toronto, Canada; 8 Department of Public Works, City of Winnipeg, Winnipeg, Canada

**Keywords:** Cycling, Build environment, Ischemic heart disease, Hypertension, Exercise, Active transportation

## Abstract

**Objective:**

To determine if expansion of multi-use physical activity trails in an urban centre is associated with reduced rates of cardiovascular disease (CVD).

**Methods:**

This was a natural experiment with a difference in differences analysis using administrative health records and trail-based cycling data in Winnipeg, Canada. Prior to the intervention, each year, 314,595 (IQR: 309,044 to 319,860) persons over 30 years without CVD were in the comparison group and 37,901 residents (IQR: 37,213 to 38,488) were in the intervention group. Following the intervention, each year, 303,853 (IQR: 302,843 to 304,465) persons were in the comparison group and 35,778 (IQR: 35,551 to 36,053) in the intervention group. The natural experiment was the construction of four multi-use trails, 4-7 km in length, between 2010 and 2012. Intervention and comparison areas were based on buffers of 400 m, 800 m and 1200 m from a new multi-use trail. Bicycle counts were obtained from electromagnetic counters embedded in the trail. The primary outcome was a composite of incident CVD events: CVD-related mortality, ischemic heart disease, cerebrovascular events and congestive heart failure. The secondary outcome was a composite of incident CVD risk factors: hypertension, diabetes and dyslipidemia.

**Results:**

Between 2014 and 2018, 1,681,125 cyclists were recorded on the trails, which varied ~ 2.0-fold across the four trails (2358 vs 4264 counts/week in summer months). Between 2000 and 2018, there were 82,632 CVD events and 201,058 CVD risk events. In propensity score matched Poisson regression models, the incident rate ratio (IRR) was 1.06 (95% CI: 0.90 to 1.24) for CVD events and 0.95 (95%CI: 0.88 to 1.02) for CVD risk factors for areas within 400 m of a trail, relative to comparison areas. Sensitivity analyses indicated this effect was greatest among households adjacent to the trail with highest cycling counts (IRR = 0.85; 95% CI: 0.75 to 0.96).

**Conclusions:**

The addition of multi-use trails was not associated with differences in CVD events or CVD risk factors, however the differences in CVD risk may depend on the level of trail use.

**Trial registration:**

Trial registration number: NCT04057417.

**Supplementary Information:**

The online version contains supplementary material available at 10.1186/s12966-022-01279-z.

## Introduction

The health of urban populations is intimately linked to the environment in which they live [1, 2]. Modifying or expanding attributes of the built environment that facilitate daily physical activity is an emerging strategy for supporting the health of urban populations. Attributes of the built environment that can facilitate daily physical activity [1, 2] include, but are not limited to, walkability [3], greenspace (i.e., parks) [4], and multi-use physical activity trails [5]. Some of these attributes are associated with higher neighbourhood-level daily physical activity [1, 6], and in some cases, are also associated with lower rates of cardiovascular disease (CVD)-related risk factors (eg. diabetes, hypertension) [7–13]. The causal nature of these observations is limited however as individuals self-select the areas in which they live and little experimental evidence exists to support these observations [8].

Multi-use trails are one of the fastest growing attributes of the built environment that support physical activity in large urban centres. In the Canadian context, a multi-use trail is a public path that creates an attractive transportation and leisure activity corridor for walking, running, and cycling through the built environment, that can be used up to 12 months of the year [14]. Multi-use trails are unique relative to other attributes of the built environment as they can be constructed with minimal change to the existing environment and support leisure-time physical activity and active transportation for large segments of an urban population [15]. Despite rapid expansion of multi-use trails in many urban centres there are few empirical studies investigating their impact on the health of the populations exposed to them.

Randomized controlled trials of attributes of the built environment are challenging [16]. Natural experiments are therefore the strongest methodological approach to infer a causal association between attributes of the built environment and health outcomes within a population [17]. Previous natural experiments of attributes of the built environment that support physical activity suffer from key methodological shortcomings [18]. First, most published experiments are at risk of selection and ascertainment biases, related to the frequent use of convenience sampling and a lack of population-level data. Second, very few experiments assessed disease-specific end-points due to limited follow-up periods [19, 20]. The current study was designed to overcome these limitations. We applied a difference-in-differences analysis to a large natural experiment to test the hypothesis that the expansion of multi-use trails within an urban setting would be associated with greater reductions in CVD-related end-points and risk factors in areas adjacent to the trails, relative to areas distant to the trails. Secondary objectives of the study included describing trail use in the 5 years following expansion and surveying trail users regarding trail use and user demographics.

## Methods

### Study design

A directed acyclic graph representing the study hypothesis and core assumptions for the analyses is presented in eFigure 1 of the appendix. We linked administrative health data that prospectively captured all deaths, hospitalizations, and drug prescriptions associated with CVD between January 1st 2000 and December 31st 2018, with census and built environment data to evaluate the natural experiment of multi-use trail expansion. The methods were published previously [21] and the a priori *hypothesis* and methods were registered at clinicaltrials.gov August 15th, 2019 (NCT04057417). The administrative health dataset provided 10 years of pre-intervention data (2000–2009) and 6 years of post-intervention data (2012–2018), with a 2-year interruption for the construction of trails (2010 and 2011). Census and built environment data were captured in 2006 for the pre-intervention period and 2016 for the post-intervention period. We followed the TREND reporting guidelines with additions outlined by Wing and colleagues for difference-in-differences studies [22]. All aspects of the study design were approved by the Biomedical Research Ethics Board at the University of Manitoba (Approval ID: REB_HS20928 (H2017232)) and the Health Information Privacy Committee within the Province of Manitoba (Approval ID: HIPC - 2019/2020–05). Participants that completed field surveys provide prior informed consent for field-based data collection.

### Study population

This study was conducted within the metropolitan area of Winnipeg, Manitoba, Canada’s seventh largest urban centre with a population of ~ 700,000 residents. Health administrative data from the Manitoba Population Research Data Repository within the Manitoba Centre for Health Policy (MCHP) were used to derive population-level estimates of CVD end-points and CVD-related risk factors as previously done [23–25]. The cohort was limited to Winnipeg residents who were aged 30 and over as of January 1, 2000 and Winnipeg residents who turned 30 years old sometime during the study period, or those older than 30 years who moved to Winnipeg sometime during the study period. All individuals were registered with Manitoba Health Insurance at least 3 years prior to January 1, 2000 or their thirtieth birthday, whichever is later, to ensure accurate health history. Individuals with a diagnosis for cystic fibrosis (ICD-9-CM code 277.0, ICD-10-CA code E84) or congenital CVD, (ICD-9-CM codes 745, 746, 747, ICD-10-CA codes Q20-Q28) at any point during the study period or 3 years prior were excluded. Individuals with existing CVD events were also excluded from the analyses. Individuals with a postal code indicating potential non-community dwelling were either excluded completely or their follow-up time was truncated when they moved to that postal code. Approximately 90% of these individuals were living in a long-term care facility or prison [3].

### Intervention: expansion of multi-use trails

**Fig. 1:**
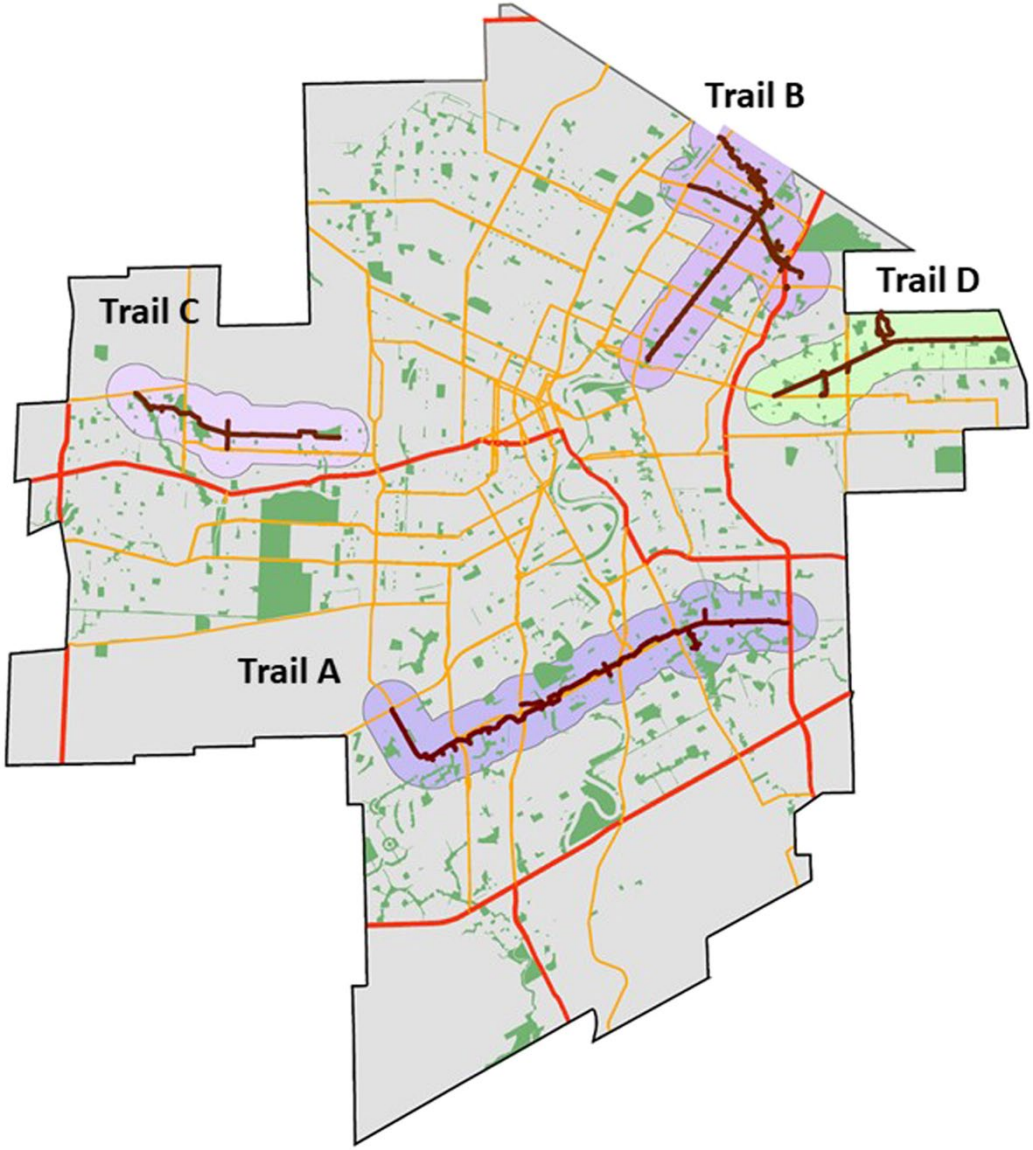
Location of multi-use trails within the City of Winnipeg. Legend: Dark lines represent actual trails and light coloured areas represent 400 m buffer for each trail. Red lines represent major roads. Orange lines represent minor roads. Green areas represent parks/greenspace

Between 2010 and 2012, the City of Winnipeg and Province of Manitoba invested $25 million to construct four paved multi-use trails, also labelled as greenways, within different areas of Winnipeg. Several mixed socio-economic dissemination areas in Winnipeg were within 400 m to 1200 m of one of the four new multi-use trails. Details of the multi-use trails are provided in the eTable 1 of the appendix and their location in Winnipeg are provided in Fig. 1. The four multi-use trails are paved two lane paths that are cleared and maintained by the City of Winnipeg, Department of Transportation 12 months of the year and cover distances of 4 to 7kms (eFigure 2). Trails were open for all forms of physical activity and accessible 24 h per day, 7 days per week, 12 months of the year with no restrictions. We considered households located within line-based buffers of 400 to 1200 m of a trail as receiving the intervention (Fig. 1). Households lying beyond the line-based buffers of 400 to 1200 m of a trail were considered comparison areas that were not exposed to the intervention. These buffer distances were based on recommendations from public partners on the team and previous studies that used buffer distances to define exposures to attributes of the built environment related to physical activity [4, 26, 27]. Individuals were assigned to the intervention or comparison conditions based on their postal code of residence, and this was updated each quarter. All individuals that met inclusion criteria and resided in a dwelling within postal code-based dissemination areas that were inside the intervention buffer were classified as receiving the intervention. All individuals residing in dwellings within postal code-based dissemination areas beyond the buffer were classified as comparison. Individuals were free to move within the City of Winnipeg and assigned to intervention and comparison dissemination areas following each move. Once an individual moved outside of Winnipeg, their follow-up time was truncated, and they were removed from the study.

### Outcome measures

The primary outcome was a composite endpoint of incident major adverse CVD events that included, CVD-mortality, ischemic heart disease, a cerebrovascular event and congestive heart failure. The secondary outcome was a composite of incident CVD-related risk factors including hypertension, diabetes and dyslipidemia. Primary outcomes were ascertained from vital statistics database and international classification for disease (ICD) codes for hospitalizations and outpatient physician visits. Secondary outcomes were estimated from validated algorithms that include physician visits, hospitalizations and outpatient prescription dispensations [24, 28]. For all three risk factors, an event was defined as one ICD-9 or ICD10 coded hospitalization or two or more recorded physician visits that included treatment for the disorder over a period of 3 years [25]. For diabetes [29] and dyslipidemia [25, 26], two or more prescriptions for a glucose or lipid lowering agent respectively were also used to define an event**.** The ICD codes used to ascertain outcomes are provided in eTable 2 and the definitions for each outcome within the composites are provided in eTable 3. Incidence rates were measured per 1000 person-years at risk.

#### Co-variates and sources of confounding

##### Socioeconomic status, ethnicity and gentrification

The average age, household income, education levels, unemployment rates, and the proportions of women, recent immigrants (within the last 10 years) and of visible minorities were calculated from the 2006 (pre-intervention) and 2016 (post-intervention) Canadian Census. We also calculated an area-level index of socio-economic status (socio-economic factor index, SEFI) for each census dissemination area, each year, that reflects neighbourhood-level social and material deprivation [29]. Gentrification was estimated from changes in property value between 2008 and 2018, assessed by the City of Winnipeg.

##### Built environment determinants of physical activity

Attributes of the built environment that support physical activity were ascertained from The Canadian Urban Environmental Health Research Consortium (CANUE) database, which was linked to health administration data [30]. From the CANUE dataset we estimated several sources of the built environment that could introduce confounding using the 2006 (pre-intervention) and 2016 (post-intervention) ArcGIS surveys. The key variable that was extracted from the CANUE database was the CANUE active living index (CAN-ALE index), which estimates the walkability (or active living friendliness) of the postal code according to the dwelling density, intersection density, points of interest and transit stops [31]. Neighbourhood-level distance to greenspace and density of recreation centres were derived from publicly available City of Winnipeg Open Data Portal.

#### Trail use and user profiles

##### Trail usage

Between June 1st 2014 and December 31st 2018, data for trail use by cyclists was obtained using Eco-Counter Dual Inductive Loop Zelts [32]. Zelts were selected as they provide an estimate of trail use that is more accurate and reliable than infrared censors, which capture both cyclist and pedestrian use but are subject to noise and less reliable in poor weather [33]. The Zelt included a Global System for Mobile (GSM) transmission device and counting unit buried on the outer edge of a trail. Zelt counters were placed at 10 locations across the four trails and collected cyclist data for 24 h/day, 7 days per week. The accuracy of the closed loop Zelt counters is ~ 90% along the trails used for this study [34].

##### Field data collection to survey trail users

To determine trail user demographics, we conducted two waves of intercept surveys among a convenience sample of 853 trail users, in 2018 and 2019. Users were surveyed while using one of the four trails and asked to complete a brief survey to provide self-reported trail usage and the perceived impact of usage on both their physical and mental health. Users also provided demographic data including self-identified gender and ethnicity, age group, newcomer status, annual household income and the first three digits of their postal code to geo-map the areas where they reside relative to the trail on which they were surveyed.

**Patient and Public involvement:** Members of the public were involved at all stages of the study. Members of the public were included through a partnership with a local non-for-profit organization (Winnipeg Trails Association) dedicated to supporting urban trails for physical activity. A member of their executive was included as a researcher on the team and members of this organization were involved in designing the study, securing funding, collecting data, and interpreting the results. Members of this organization also helped recruit trail users during field data collection and collected survey responses. Lastly, this non-for-profit organization co-developed public and policy maker-friendly tools to disseminate results of the study via their social media platforms and at local meetings.

### Statistical analyses

**Sample Size Calculations**: Minimal detectable effect sizes were calculated assuming clustered count data, where treatment assignment was at the dissemination area and the outcome of interest was an incidence rate of a CVD-related event or risk factor (e.g. Poisson count) [34]. Using pre-intervention neighbourhood-level incidence rates of ≈ 49.5 ± 4.55 per 1000 person years [23], an alpha = 0.05, and a beta = 0.2, we were powered to detect a 10% difference in the primary outcome with 50 intervention dissemination areas and a 9% difference with 60 intervention dissemination areas [35].

**Hypothesis Testing:** First, we compared geographic and descriptive variables between dissemination areas (defined by postal codes) within 400 m of a new multi-use trail and outside 400 m of the trail, using t-tests. Next, we calculated crude rates of the primary and secondary outcomes for each quarter of the study years separately for intervention and comparison dissemination areas with Poisson regression models and included the trail exposed or unexposed person-time at risk in each quarter as an offset. We tested the parallel trends assumption by examining time trends for the main outcome measures in both the intervention and comparison dissemination areas prior to the trail expansion.

To calculate propensity scores, we conducted three area-level logistic regression models (i.e., using 400 m, 800 m and 1200 m buffers) predicting exposure to the intervention. Covariates used to calculate the propensity for a neighbourhood being in the intervention condition average age, household income, education levels, unemployment rates, and the proportions of women, recent immigrants (within the last 10 years) and of visible minorities, percentage of active commuters, mean SEFI, distance to greenspace, density of recreation centres and neighbourhood walkability score. Predicted probabilities of exposure to the intervention (propensity scores) were used to match exposed (urban trail) to unexposed dissemination areas (outside the 1200 m buffer) at a 1:3 or 1:2 ratio, with dissemination areas outside the a priori caliper of ±0.05 excluded. Standardized differences were calculated before and after matching. An individuals dwelling was used to assign them to intervention or comparison dissemination areas for each quarter.

We conducted difference-in-difference analyses comparing incident rates of CVD events and CVD risk factors between intervention and comparison areas, stratified by a 400 m buffer from a trial, before and after the introduction of a trail using in propensity score matched Poisson and negative binomial regression models. These were repeated for 800 m and 1200 m buffers. Models included an indicator for exposure to a trail (intervention or comparison), an indicator for the time period (pre-post intervention), and an interaction term between urban trail expansion and the time period (difference-in-differences). Individuals were clustered into age, sex, SEFI and Charlson index strata, which were the unit of analysis for the propensity score matched analyses.

**Sensitivity analyses:** We repeated the regression analyses above with intervention buffers set at 800 m and 1200 m. We also repeated propensity score matched regressions for (A) males and females separately; (B) with a cohort restricted to individuals aged 30–65 years, (C) for each trail separately and their matched comparison areas, and finally (D) for outcomes that occurred in summer (April–September) and winter (October–March) months separately.

Data are presented as means with SDs or 95% CIs. All *P* values were 2-sided, and P values of 0.05 or less were considered statistically significant. Analyses were conducted using SAS version 9.4 (SAS Institute).

## Results

### Demographics of intervention and comparison areas

Demographic variables stratified by intervention and comparison areas are presented in Table 1. Using census data from 2006 to represent the pre-intervention time period, 107 dissemination areas were within the 400 m intervention buffer (intervention areas) and 1055 dissemination areas were outside the 400 m buffer (comparison areas). Prior to the intervention, the annual number of residents 30 years and older that did not have a CVD event at the time of cohort entry was 314, 595 (IQR: 309,044 to 319,860) in the control group and 37,901 residents (IQR: 37,213 to 38,488) in the intervention group. During the pre-intervention time period, mean age, percentage of women, percentage of the residents without high school completion and average property value were similar in comparison and intervention areas (standardized differences < 0.15) (Table 1). Comparison areas had a greater percentage of recent immigrants, visible minorities, a lower SEFI score, and higher household income and were further away from greenspace (all standardized differences > 0.2). These variables remained stable throughout the full study period in both intervention and comparison areas. Comparison areas experienced greater gains in household income and average property values than intervention areas over the 18-year study period. After propensity score matching, standardized differences for all variables were less than 0.08, except for differences in walkability (0.15) and the percentage of the population that reported active commuting (0.12) (Table 1). Demographic variables for each individual trail are provided in eTable 4.

**Table 1:** Dissemination area demographics in intervention and control dissemination areas

Variable	Control Areas		Intervention Areas		Standardized Differences	
	2006	2016	2006	2016	Crude	PS-Matched
Dissemination Areas^a^ (DA)	1055	1088	107	98	–	–
N^b^	356,237	416,381	42,853	47,591	–	–
Female^b^ (%)	52.14%	51.64%	53.37%	52.84%	0.06	0.003
Age^b^ (years)	52.57	53.14	52.76	54.16	0.08	0.006
Visible Minority^a^ (%)	15.22%	24.28%	10.87%%	17.95%	0.37	0.06
Immigrated last 10 years^a^ (%)	4.84%	11.23%	3.83%	8.22%	0.29	0.06
Socioeconomic indicators						
SEFI^b^	−0.2677	−0.2834	−0.3673	−0.2331	0.26	0.004
Household income^a^	$66,497	$91,199	$71,758	$92,825	0.15	0.06
Average Property Value^c^	$118,786	$311,553	$124,944	$307,819	0.02	0.004
Population without high school graduation^a^ (%)	23.40%	17.47%	22.40%	16.86%	0.11	0.003
Unemployment Rate^a^	5.28%	6.74%	5.24%	6.10%	0.16	0.007
Activity indicators						
Fitness/Recreation Centres within 5km^c^ (2018 only)	N/A	84.56%	N/A	95.35%	–	–
Average Distance to greenspace^c^ (m)	268.51 m	238.49 m	157.77 m	127.22 m	0.21	0.007
Walkability Score^d^	0.6068	0.7494	0.1165	0.2747	0.46	0.15
Active commuting^a^ (%)	8.29%	7.32%	5.03%	4.31%	0.44	

^a^Census (based on all DAs in 400 m buffer)

^b^MB Health Insurance Registry (based on total Winnipeg population age 30+, Dec 31 2006 & 2016, 400 m buffer)

^c^City of Winnipeg Open Data Portal

^d^CANUE (ALE_06 = total of all Z scores, 400 m buffer)

### Stable unit value treatment assumption

Over the 18-year study period, 44.8% of individuals changed postal codes. To assess possible spillover effects from residential mobility, we first calculated the number of individuals that moved from the last year of the pre-intervention period (2009). We found that 95%, 92% and 90% of the population remained in their assigned treatment arms during the follow-up period at 400 m, 800 m and 1200 m buffers respectively. Using the 400 m buffer, 95.2% of the study population remained in their pre-intervention study arm during the follow-up period, 2.5% moved from the control to the intervention areas and 2.3% moved from the intervention area to a control area. Among the 4.8% of individuals that moved from their assigned neighbourhood area during the study period, they were younger (50 vs 54 yrs), more likely to be in the upper quintile of socio-economic status (23% vs 16%) and had lower weighted sum of Charlson comorbidities (0.39 vs 0.44), compared to individuals that remained in their assigned study area during the follow-up period. Importantly, individuals that moved from the control area to an intervention area were similar to individuals that moved from an intervention area to control areas on several co-variates, including age (50.4 ± 16.7 vs 50.8 vs 16.6 yrs), percentage of the group in the upper quintile of household income (23.2 vs 23.7%) and sum of weighted Carlson comorbidity indexes (0.32 ± 0.67 vs 0.32 ± 0.66).

### Trail use and user characteristics

**Fig. 2:**
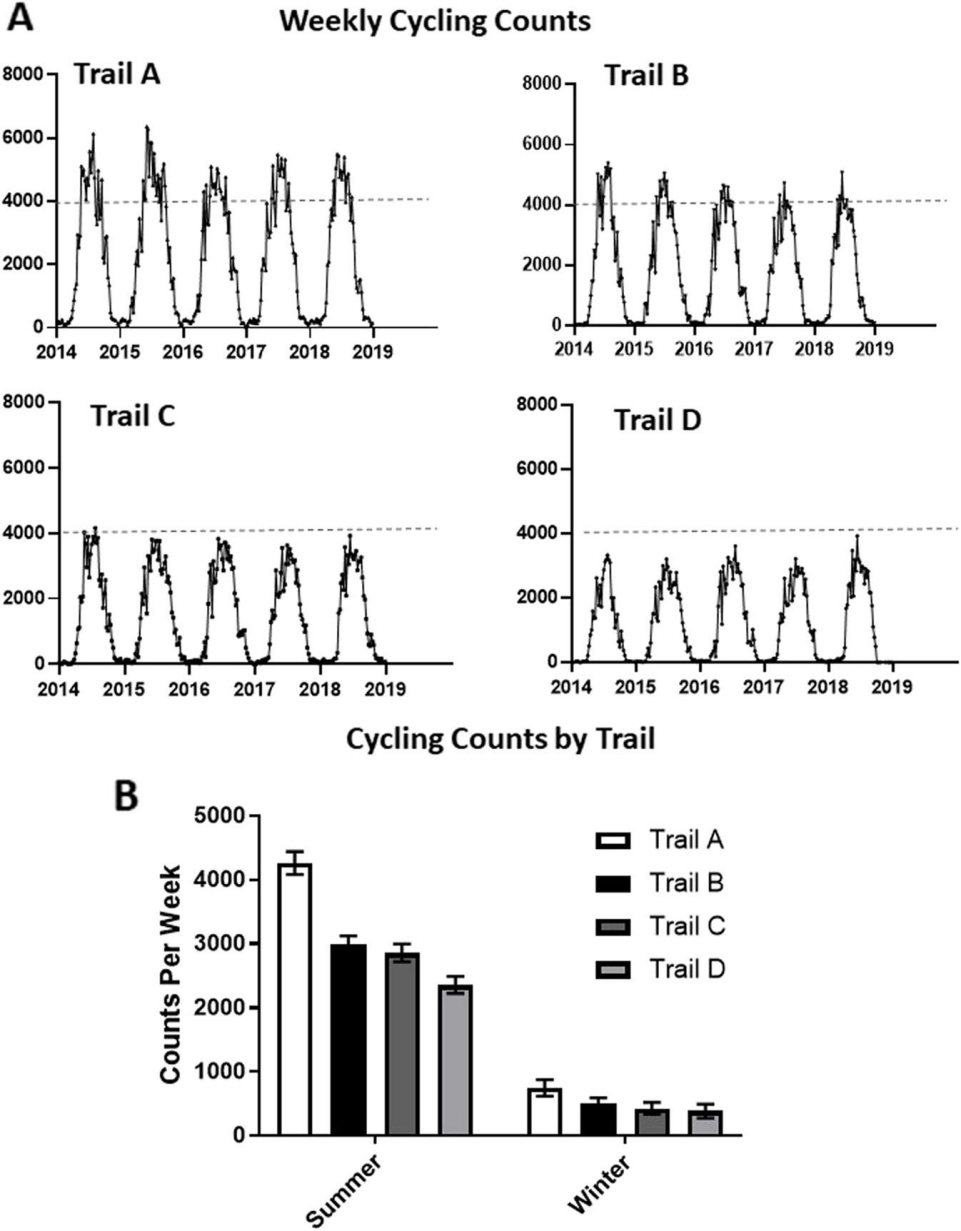
Trail use following construction of multi-use trails. **A-D**. Weekly unidirectional cycling counts over 5 years for each of the four trails studied; **E** – Trail counts in summer and winter months for each trail. Bars = mean, error bars = 95% confident intervals

The four new multi-use trails ranged from 4kms to 7kms in length and were constructed within largely suburban areas (Fig. 1). Images before and after trail construction are provided in the appendix (eFigure 2). Between January 1st 2014 and December 31st 2018, 1,681,125 bi-directional bicycle counts were recorded on all four trails. Trail use varied across the four trails, with highest bicycle counts captured on the longest trail with the highest mixed land use, and lowest bicycle counts captured on the shortest trail located largely in a suburban neighbourhood with the lowest mixed land use (Fig. 2A-D). Counts were approximately 6-fold higher during spring and summer months (May–September) than other months of the year (Fig. 2E). Compared to weekdays, weekend days were characterized by lower trail counts (707 vs 809 counts per day, *p* = 0.02). Hourly trail counts were highest in the morning and evening hours, compared to mid-day. Trail user profiles are provided in eFigure 3 in the appendix. Most users surveyed were 30-65 yrs. of age, had an annual household income over $75,000 (CAN) and identified as white-Caucasian. Over 85% of trail users reported travelling less than 15 min to access the trail in their neighbourhood (eFig. 3D).

### Major adverse CVD events and CVD risk factors

Crude rates of CVD events and CVD risk factors in the pre- and post-intervention time periods for both intervention and comparison areas are provided in Table 2. There were 51,316 CVD events and 135,720 CVD risk events in the pre-intervention time-period and 24,012 CVD events and 47,608 CVD risk events in the post-intervention time-period. The proportion of the composite outcome of CVD events attributed to CVD deaths (14.7 vs 12.2%), incident ischemic heart disease events (46.6 vs 43.8%), incident cerebral infarctions (8.8 vs 11.0%) and incident cases of heart failure (29.7 vs 32.9%), were similar in the pre- and post-intervention periods. The proportion of the composite outcome of CVD risk factors attributed to hypertension (42.6 vs 40.4%), dyslipidemia (40.1 vs 37.4%) and diabetes (17.1 vs 22.2%) was also similar in the pre- and post-intervention time periods. Between the pre- and post-intervention time periods, annual composite CVD event rates declined in both the comparison (15.5 per 1000 persons; 95%CI: 15.4–15.7 per 1000 persons vs 10.7 per 1000 persons; 95%CI: 10.6–10.9 per 1000 persons; pre-post difference = − 4.8 per 1000 persons) and intervention areas (15.0 per 1000 persons; 95%CI: 14.7–15.3 per 1000 persons vs 11.5 per 1000 persons; 95%CI: 11.2–11.8 per 1000 persons; (pre-post difference = − 3.5 per 1000 persons). Composite CVD risk factor rates also declined in both the comparison (48.0 per 1000 persons; 95% CI: 47.9–48.3 per 1000 persons vs 32.1 per 1000 persons; 95% CI: 31.7–32.4 per 1000 persons; pre-post difference = − 15.9 per 1000 persons) and intervention areas (48.7 per 1000 persons; 95% CI: 47.7–48.3 vs 32.5 per 1000 persons; 95% CI: 31.8–33.1 per 1000 persons; pre-post difference = − 16.2 per 1000 persons) between the pre- and post-intervention time periods.

**Table 2:** Crude rates of major adverse cardiovascular events and cardiovascular disease risk (CVD) factors

Crude	Control Neighbourhoods		Intervention Neighbourhoods	
	2000–2009	2012–2018	2000–2009	2012–2018
Median Population per quarter (IQR)	314,595 (309,044-319,860)	303,853 (302,843-304,465)	37,901 (37,213-38,488)	35,778 (35,551-36,053)
*CVD Events*				
Cases of CVD endpoints	40,826	18,702	10,490	5310
Incidence per 1000 person-years at risk (95% CI)	15.5 (15.4–15.7)	10.7 (10.5–10.9)	15.0 (14.7–15.3)	11.5 (11.2–11.8)
*CVD Risk Factors*				
Cases of CVD risk factors	107,008	37,652	28,712	9956
Incidence per 1000 person-years at risk (95% CI)	48.0 (47.7–48.3)	32.1 (31.7–32.4)	48.7 (48.2–49.3)	32.5 (31.8–33.1)

*CVD* cardiovascular disease; IQR = inter-quartile range; 95% = 95% confidence intervals

### Difference-in-differences estimates

**Fig. 3:**
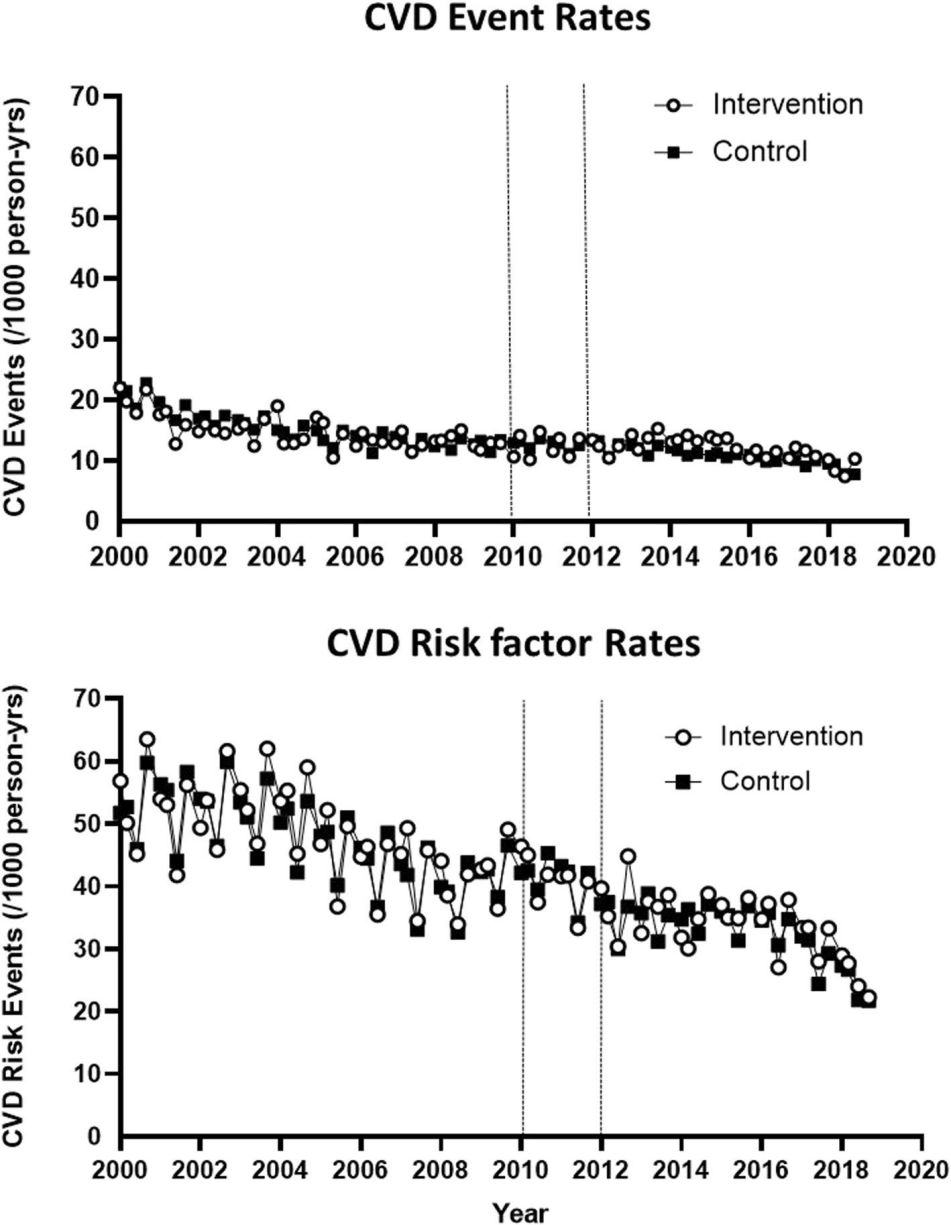
Crude quarterly incident rates for the composite measures of Cardiovascular Disease (CVD) Events and Risk Factors over time for intervention and comparison areas

The seasonal rates of CVD events and CVD risk factors for intervention and comparison areas throughout the 18-year time frame of the natural experiment are presented in Fig. 3. In propensity-score matched analyses, the parallel trends assumption was met in the pre-intervention time period, with similar rates of decline in incident CVD events (β = 0.05, 95% CI: − 0.19, 0.28, *p* = 0.68) and CVD risk factors (β = − 0.001, 95% CI: − 0.14, 0.11, *p* = 0.88) in both intervention and comparison areas, respectively (eFigure 4).

**Fig. 4:**
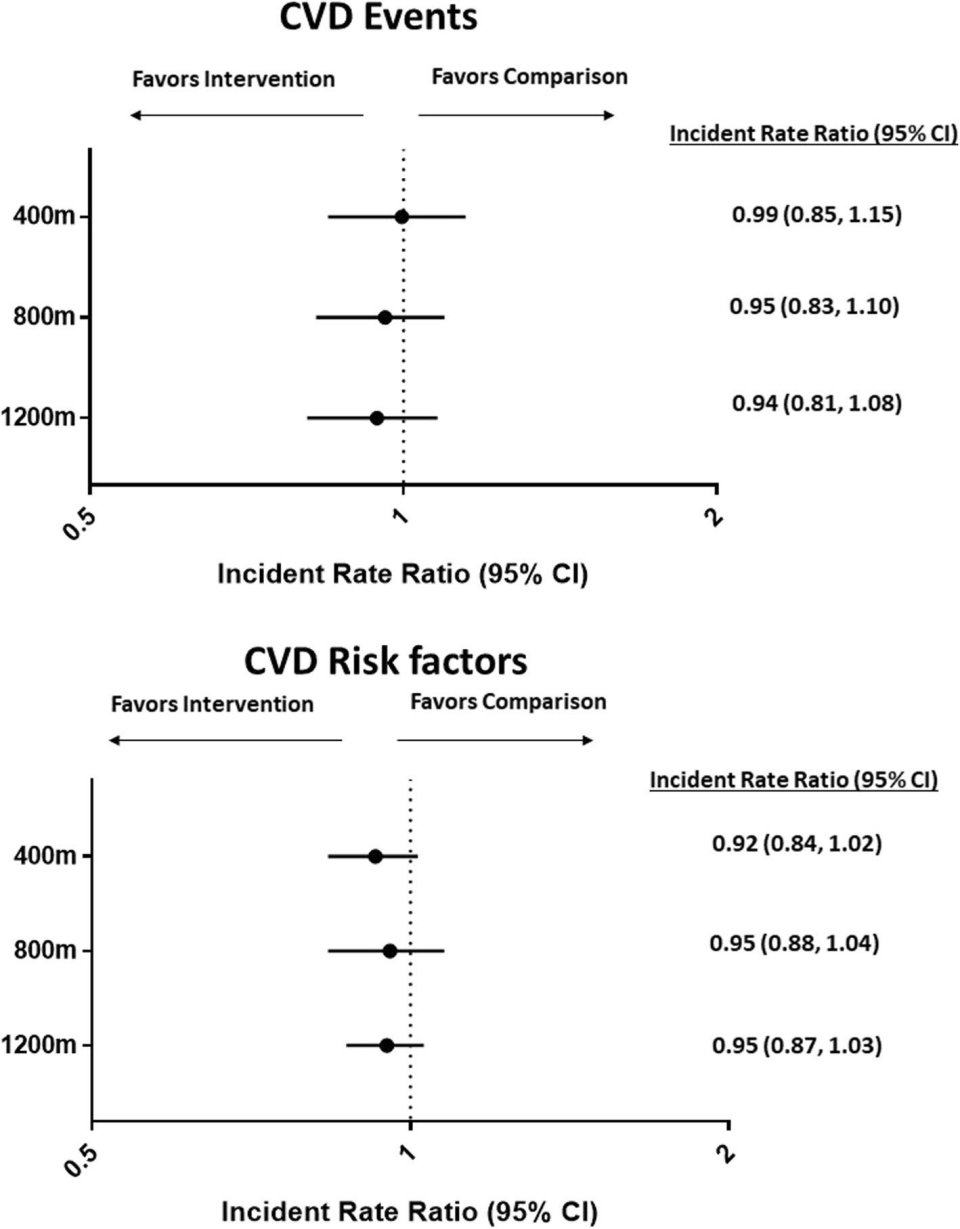
Difference-in-differences for CVD events and CVD Risk Factors across different intervention buffers

The results of the difference-in-differences estimates for primary and secondary outcomes in the propensity score matched analyses are presented in Fig. 4. After propensity score matching, the incident rate ratio for the intervention relative to the comparison areas, using a 400 m buffer, was 1.06 (95% CI, 0.90, 1.24, *p* = 0.51). Propensity score matched incident rate ratios for CVD events remained similar when the intervention buffer was increased to 800 m (0.99; 95% CI: 0.85, 1.14, *p* = 0.89) and 1200 m (0.95; 0.82, 1.10, *p* = 0.49). After propensity score matching, the incident rate ratio for CVD risk factors in the intervention relative to comparison areas, using the 400 m buffer, was 0.92 (95% CI, 0.84, 1.02; *p* = 0.10). Propensity score matched incident rate ratios for CVD risk factors remained similar when the intervention buffer was increased to 800 m (0.95; 95% CI: 0.88, 1.04, *p* = 0.26) and 1200 m (0.95; 0.87, 1.03, *p* = 0.21) (Fig. 4).

### Sex-specific and sensitivity analyses

**Fig. 5:**
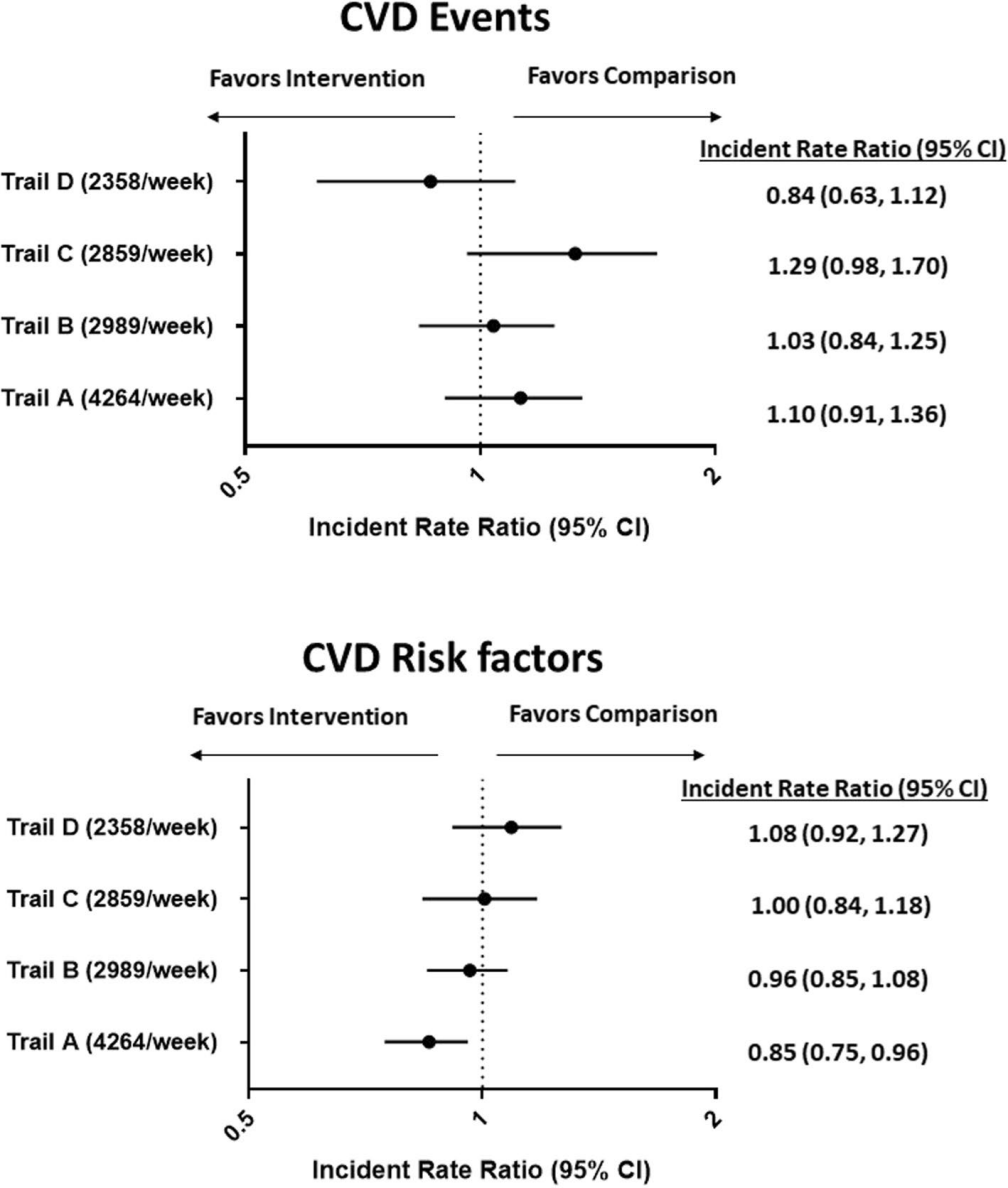
Difference-in-differences for CVD events and CVD Risk Factors by trail. Legend: Numbers in brackets beside each trail reflect mean weekly cyclist counts during the summer months

Descriptive factors for each trail are provided in eTable 4. The trail with the highest use (Trail A), was characterized by higher median household income, higher property values, a larger proportion of citizens that reported being a visible minority and less greenspace, relative to trails with less use by cyclists. Results from separate propensity score matched difference in differences analyses for each multi-use trail are presented in Fig. 5. In propensity score matched analyses, no differences were noted for CVD events across all four trails; yet rates of CVD risk factors were significantly lower in areas within the 400 m (incident rate ratio: 0.85; 95% CI: 0.75, 0.96), 800 m (incident rate ratio: 0.86; 95% CI: 0.77, 0.96), and 1200 m (incident rate ratio: 0.88; 95% CI: 0.80, 0.98) of Trail A, the longest trail with the highest weekly cycling counts, compared to comparison areas. These trends were not observed for the other three trails (B-D), which were shorter and displayed lower cycling counts (Fig. 5). To test for possible spillover effects from individuals that moved into intervention areas during the study period, we repeated the propensity score matched regression analyses restricted to individuals that did not move during the study period. When analyses were restricted to those who did not move during the study period the effect size and precision of the estimate for primary (full sample IRR: 1.06, 95% CI: 0.90 to 1.24 vs non-movers IRR: 1.04, 95% CI: 0.88, 1.22) and secondary outcomes (full sample IRR: 0.92; 95% CI: 0.84 to 1.02 vs non-movers IRR: 0.92; 95% CI: 0.84, 1.01) were very similar.

When sex and intervention were added as an interaction term, we did not observe sex-specific differences in the effect of trail exposure on primary or secondary outcomes. Sex-specific propensity scored matched regression models are presented in eFigure 5. The differences in CVD events and CVD risk factors observed for the entire population were similar for both males and females. Restricting analyses to the urban population between 30 and 65 years of age did not influence the strength of the difference-in-difference estimates for CVD events, however the effect size for CVD risk factors was marginally greater that the estimate when the entire population was included in the analyses (incident rate ratio: 0.89; 95% CI: 0.77, 1.02; eFigure 6). Restricting analyses to CVD events and risk factors that occurred in summer and winter months separately, yielded estimates that were similar to those observed using the full dataset (eFigure 7).

## Discussion

To our knowledge, this is the largest natural experiment to date examining the impact of a change to the built environment that facilitates physical activity on CVD-related events and risk factors. We found that the construction of four paved multi-use trails affected ~ 48,000 individuals living within 400 m of a trail and was associated with frequent use by cyclists that was sustained for up to 5 years. Using a difference-in-differences analysis, we observed no significant differences in CVD events or CVD risk factors in the dissemination areas adjacent to a newly constructed trail compared to areas that were more distal to a new trail over the 6-year follow-up period. However, in sensitivity analyses assessing effects for each of the four trails separately, we observed a 12–15% lower rate of CVD risk factors in areas within 400-1200 m of the multi-use trail with the highest frequency of trail use, compared to the areas outside this buffer. These data provide unique experimental evidence that an expansion of multi-use trails may be associated with a reduction in CVD risk factors in areas adjacent to a trail, however this effect may be sensitive to frequency of trail use, or trail characteristics.

A core assumption when creating attributes of the built environment that support physical activity is that citizens will use them. Previous natural experiments reveal that expanding multi-use trails in an urban setting increase trail use and physical activity levels of individuals [36–38]. For example, following the creation of a 3-mile paved multi-use trail in Knoxville Tennessee, areas adjacent to the trail observed a 3-fold increase in the number of pedestrians and cyclists, compared to a comparison areas that were not exposed to a new trail [37]. The increase in trail use following the expansion of a recreation trail is quite variable however, ranging from 30 to 1200 users per day [39, 40]. We found that trail use by cyclists ranged between 1800 to 5000 per week during summer months, and this intensity of trail use remained consistent for 5 years. Trail use varied two-fold across the four new trails, with the highest use observed on the longest trail constructed in areas with higher mixed-land use. This is an important area for future investigation, and for future policies related to aspects of the built environment that support physical activity, as a gradient in CVD-risk factor rate differences was  observed according to the magnitude of weekly trail use.

Attributes of the built environment that support physical activity are associated with better health outcomes for individuals living in areas adjacent to them. Several population-based observational studies indicate that individual living in areas with more greenspace, or higher walkability experience 5–10% lower rates of CVD events [4] or CVD-related risk factors [3, 12], compared to areas with less greenspace or walkability. For example, among ~ 24,000 adults in the UK with a mean age of 59.2 years, the risk for CVD events was 3% lower (95% CI: 0.96, 0.99) among those who lived in areas with the highest amount of greenspace, compared to those with the least amount of greenspace [4]. Conversely, in a cross-sectional study of more than 44,000 adults with a mean age of 54 years, the odds of CVD risk factors were 10–15% higher among those residing in the least walkable areas, compared to those in the most walkable areas [12]. The findings from the current study expand on these observations by describing changes in CVD-related health outcomes following a large change in the built environment that supports daily physical activity. Using an experimental difference-in-differences analyses coupled with propensity matching techniques and a 400 m buffer to define intervention areas, we did not find a difference in CVD events however CVD risk factor rates were 8% lower (incident rate ratio: 0.92; 95% 0.84 to 1.02) in areas exposed to a multi-use trail compared to matched unexposed comparison areas. This effect was most pronounced in areas adjacent to the trail with the highest use by cyclists (incident rate ratio: 0.85; 95% CI: 0.75 to 0.96) and the effect decreased in a dose-response manner along trails with lower trail use. The trail with the highest cycling use and the largest reductions in CVD risk occurred within areas with higher mixed land use (eTable 4). While we propensity-score matched for several measures of built environment, it is possible that certain aspects of the mixed land use, could contribute to the observed effects. These experimental data expand the reported associations in observational studies and have potential policy implications as trail use may be important determinant of CVD-related health benefits associated with new trails.

The results presented here are strengthened by several factors. First, several assumptions for using difference-in-differences analyses [22] were satisfied, including (1) parallel trends in outcomes prior to the intervention; (2) the observation that the construction of the trails was unrelated to the outcomes of interest at baseline (i.e. exogeneity), and there were no common causes of building the trails and CVD-related outcomes at baseline and (3) the composition of the population within the intervention and comparison areas remained stable over the 18 years of the study. Second, the main end-points of interest were available for the entire urban population over a period of 18 years, limiting the risk of ascertainment bias. Lastly, linking administrative health data with census and built environment data allowed us to capture significant sources of confounding and build robust propensity scores to match intervention and comparison areas. Despite these strengths, there are several limitations that need to be addressed. First, we do not have information on individual-level physical activity, individual-level trail use, or the percentage of the population in each neighbourhood that used the trail. Accordingly, the extent to which the trails increased physical activity levels among individuals living in areas adjacent to the trails is unclear. Additionally, without individual physical activity levels we cannot conclude that any effects observed here were directly related to changes individual-level physical activity levels. The urban centre studied was relatively small, reducing the generalizability of the findings. Second, the natural experiment occurred in an urban centre with a longer and colder winter season, which was associated with significant reductions in trail use, potentially limiting the impact of the intervention and the generalizability of these findings. Third, we are unable to control for selection bias, as individuals self select the areas in which they live and active individuals may self-select areas that have better access to urban trails. Forth, outcomes were captured using algorithms that rely on ICD-codes, physician billing records and drug prescriptions. These algorithms have been validated and have excellent specificity but only modest sensitivity. Lastly, it is possible that individuals in comparison areas travelled to and used the multi-use trails, increasing the risk of “contamination effects” from the intervention to the comparison areas. This bias is less concerning however, as any significant contamination of the intervention by individuals from comparison areas would only bias our results towards the null. Additionally, the use of three different line-based buffers to define the intervention and survey data from trail users suggest that if there was contamination, the influence was minimal.

## Conclusions

The addition of four paved, multi-use trails in an urban setting was associated with significant and sustained use by cyclists for 5 years following construction. The expansion of the trails was not associated with differences in CVD-related events or CVD risk factors overall, yet a modest difference in CVD risk factors were observed in areas adjacent to the trail with the highest weekly use by cyclists. The effect of an expansion of multi-use trails on CVD-risk factor reduction may depend on proximity to trails and the magnitude of trail use.

## Supplementary Information

**Additional file 1: eFigure 1.** Directed acyclical graph depicting the study hypothesis. **eFigure 2.** Trail areas before and after the intervention. **eFigure 3.** Trail user profiles. **eFigure 4.** Parallel trends in CVD events and CVD risk factors prior to the intervention. **eFigure 5.** Sex specific effects of multi-use trails on CVD events and CVD risk factors. **eFigure 6.** Effects of multi-use trails in CVD events and CVD risk factors for the population restricted to 30–65 years. **eFigure 7.** Effects of multi-use trails on CVD events and CVD risk factors stratified by season. **eTable 1.** Details of the multi-use trails. **sTable 2.** Cardiovascular disease events and cardiovascular disease risk factor classification and corresponding International Classification of Disease (ICD) codes, in alphabetical order. **sTable 3.** Definitions and sources of data to define cardiovascular disease events and cardiovascular disease risk factors. **eTable 4.** Area-level descriptive variables for each multi-use trail.

## Data Availability

The data used in this study were obtained from the Manitoba Population Research Data Repository, housed at the Manitoba Centre for Health Policy, University of Manitoba, and were derived from data provided by Manitoba Health and the Winnipeg Regional Health Authority. These data are owned by the data providers: Manitoba Health, Seniors and Active Living, and Manitoba Families. Access to the data may be granted upon receiving approvals from the University of Manitoba Health Research Ethics Board and the Health Information Privacy Committee, along with permission from both data providers. The authors are unable to provide direct access to the data without approval from the committees and ethics board, as stated above. More information about access to these databases is available at http://umanitoba.ca/faculties/health_sciences/medicine/units/chs/departmental_units/mchp/resources/access.html. Data for trail use are owned and managed by the City of Winnipeg and require approval from the City of Winnipeg Department of Transportation to be accessed.
