# Supplementary material for: Multi-use physical activity trails in an urban setting and cardiovascular disease: a difference-in-differences analysis of a natural experiment in Winnipeg, Manitoba, Canada
Source: Int J Behav Nutr Phys Act. 2022 Mar 28;19:34. doi: 10.1186/s12966-022-01279-z (PMC8962160; doi:10.1186/s12966-022-01279-z)
Supplement: Supplementary file 1 — Additional file 1: eFigure 1. Directed acyclical graph depicting the study hypothesis. eFigure 2. Trail areas before and after the intervention. eFigure 3. Trail user profiles. eFigure 4. Parallel trends in CVD events and CVD risk factors prior to the intervention. eFigure 5. Sex specific effects of multi-use trails on CVD events and CVD risk factors. eFigure 6. Effects of multi-use trails in CVD events and CVD risk factors for the population restricted to 30–65 years. eFigure 7. Effects of multi-use trails on CVD events and CVD risk factors stratified by season. eTable 1. Details of the multi-use trails. sTable 2. Cardiovascular disease events and cardiovascular disease risk factor classification and corresponding International Classification of Disease (ICD) codes, in alphabetical order. sTable 3. Definitions and sources of data to define cardiovascular disease events and cardiovascular disease risk factors. eTable 4. Area-level descriptive variables for each multi-use trail. [file 12966_2022_1279_MOESM1_ESM.docx]

**SUPPLEMENTAL FILE**

eFigure 1. Directed acyclical graph depicting the study hypothesis


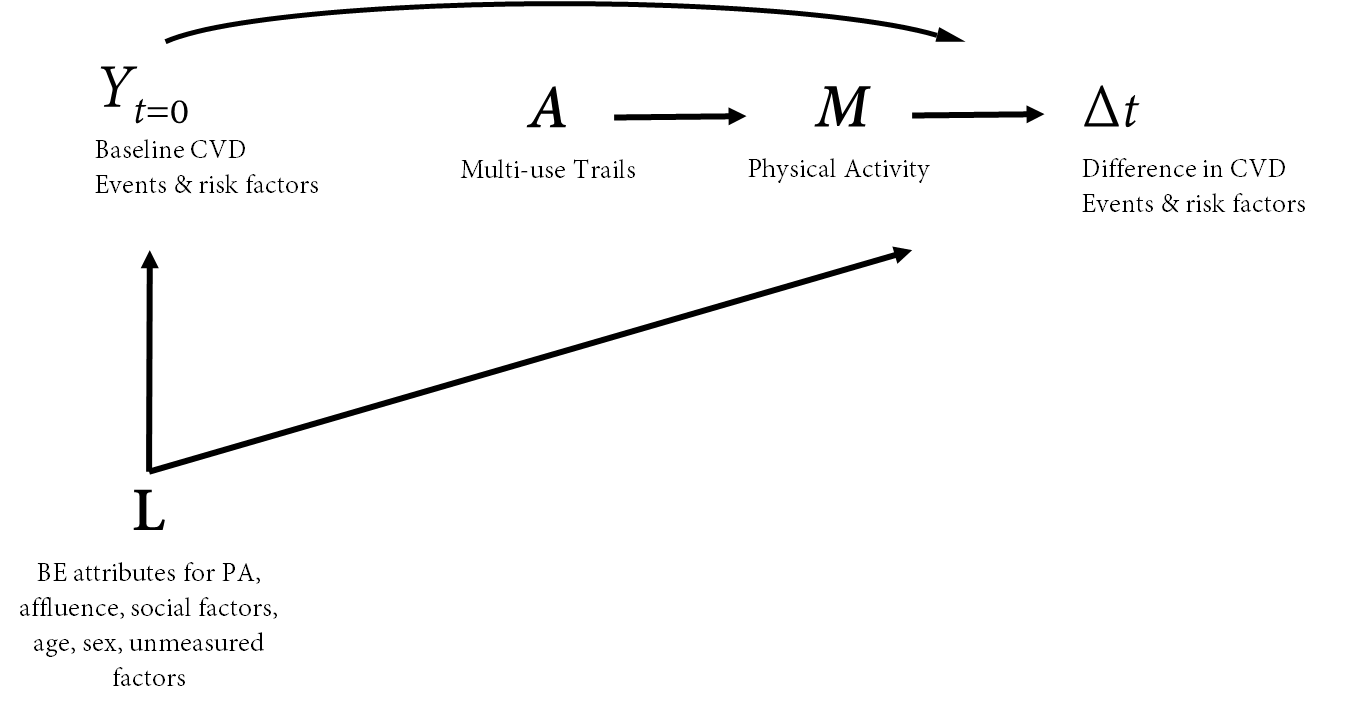


Y = outcomes of CVD events at baseline (2000-2010); A = intervention of receiving a multi-use trail (yes/no); L = measured and unmeasured factors that could influence baseline rates or changes in rates of CVD events and risk factors.

The following assumptions required for causal interpretation were included in the graph:

(1) The intervention of building multi-use trails (“*A*”) was well-defined within the City of Winnipeg (see appendix Figure 2 for visual representation) and the counterfactual of not building the recreational trails is observable;

(2) There are no common causes of building multi-use trails and the changes in CVD events/risk factors (Δ*t*);

(3) the decision to build the trails in their specific locations were unrelated to baseline levels of CVD events/risk factors (i.e. no arrow from *Y_t=_*_0_) in the city;

(4) there are no common causes of baseline CVD events/risk factors and building the recreational trails;

(5) few people moved from control areas to intervention areas and those who did, did so at random (exogeneity);

(6) any association between multi-use recreational trails and CVD events/risk factors is mediated through physical activity levels (“*M”*) of individuals within areas adjacent to the trails and

(7) the entire population of living with 400 – 1200 m of the trail had access to the recreational trail and those living beyond 400 to 1200m from the trail were less likely to use it.

eFigure 2. Trail areas before and after the intervention

**Trail A**


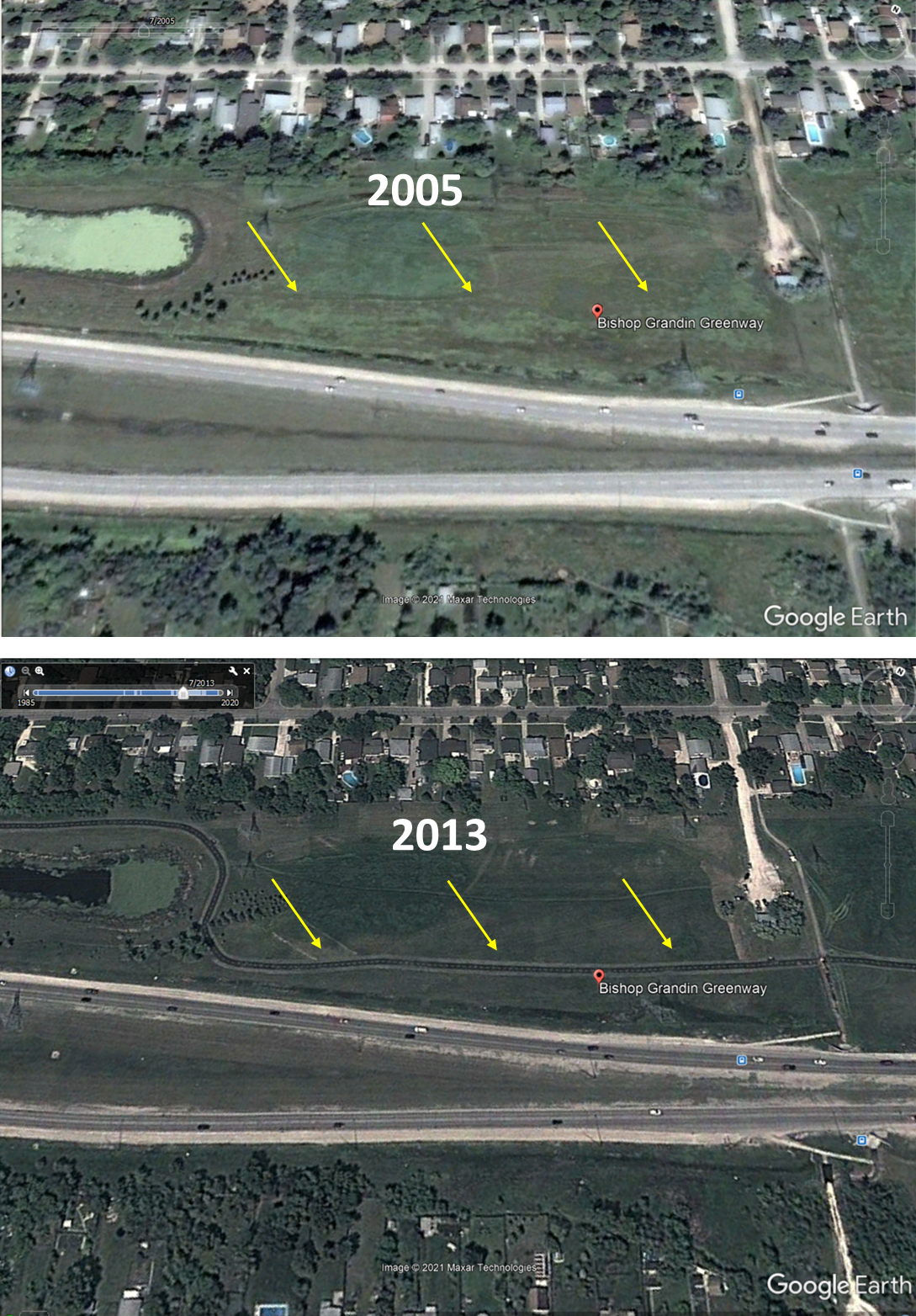


Trail B


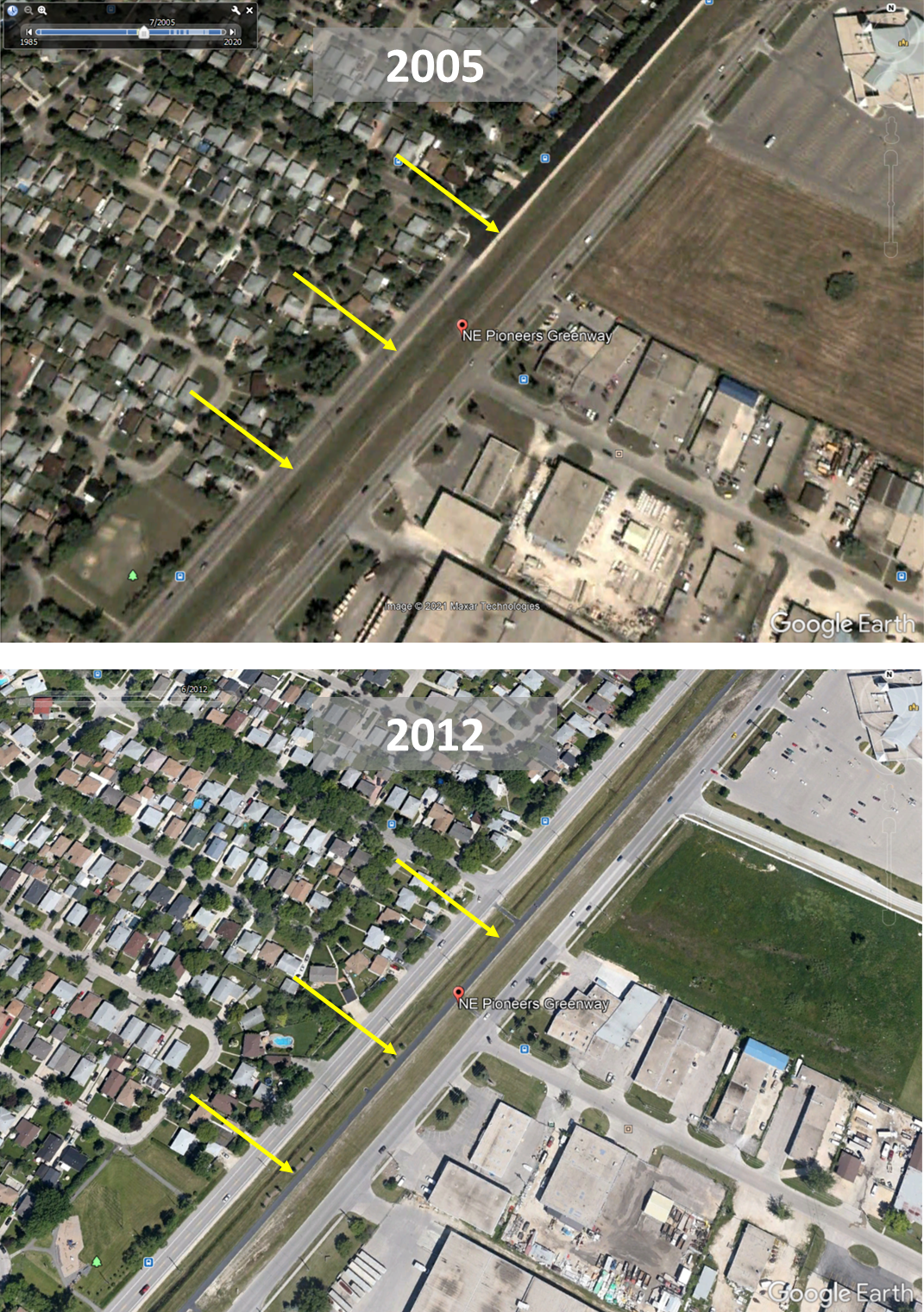


Trail C


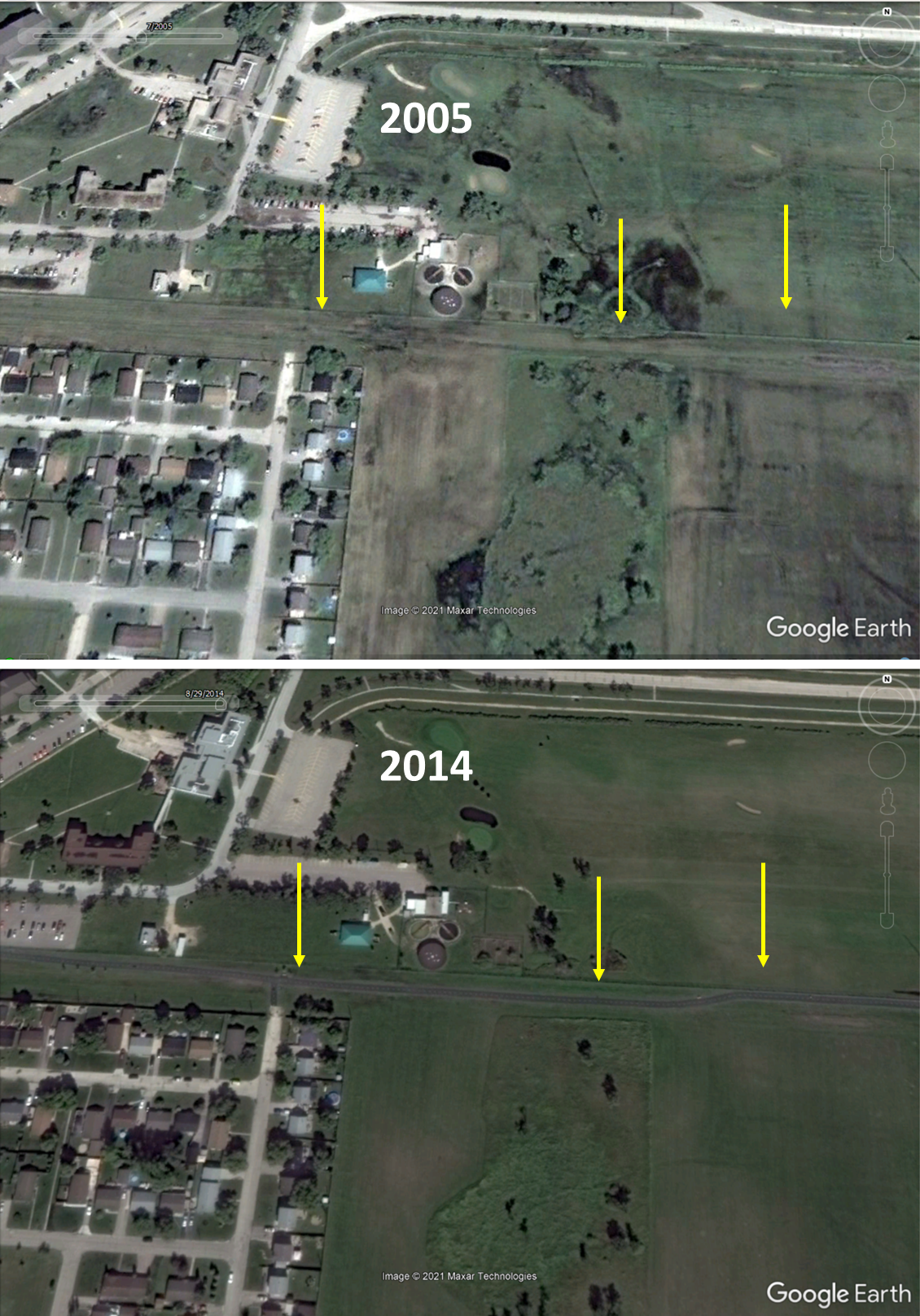


Trail D


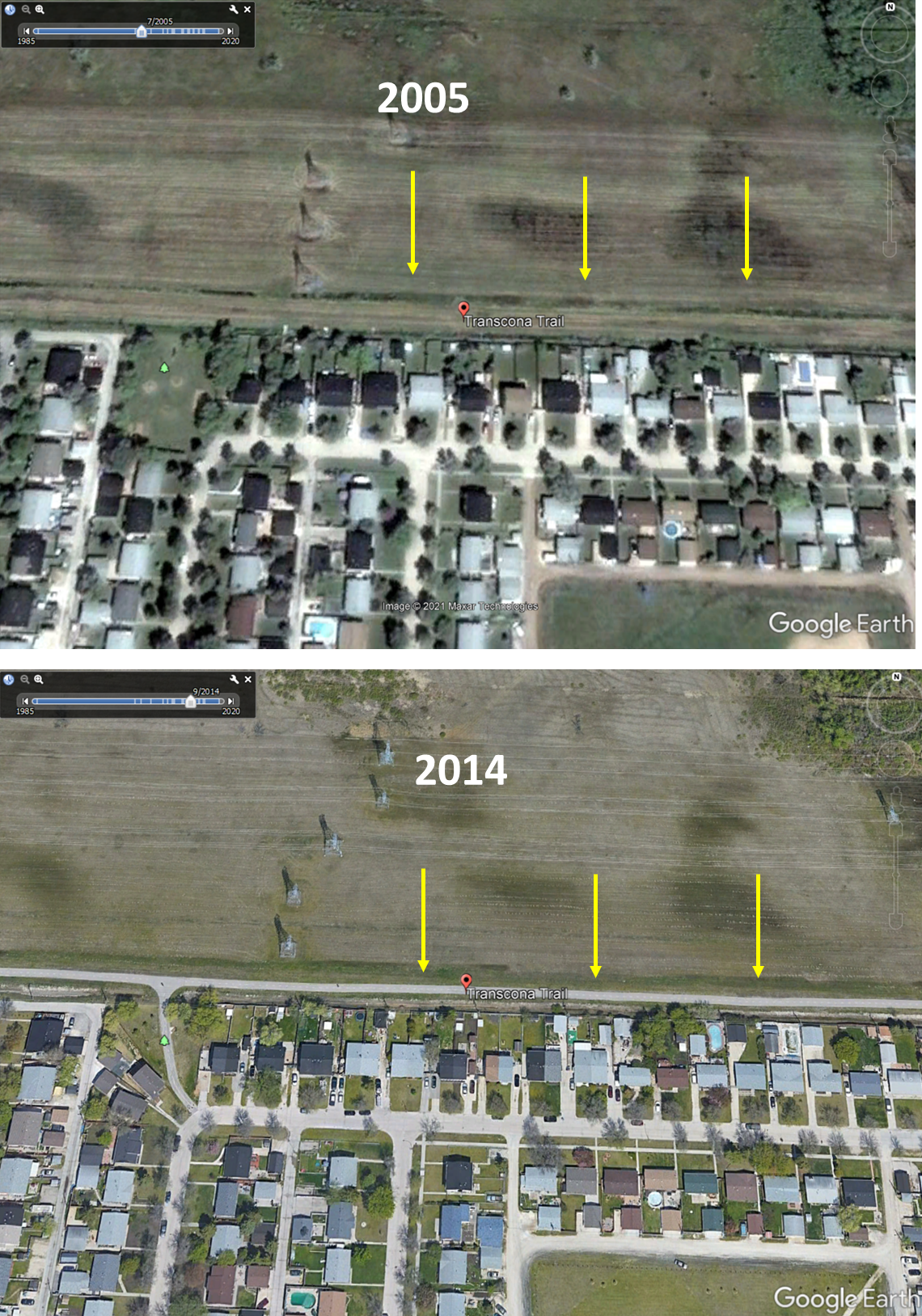


eFigure 3. Trail user profiles


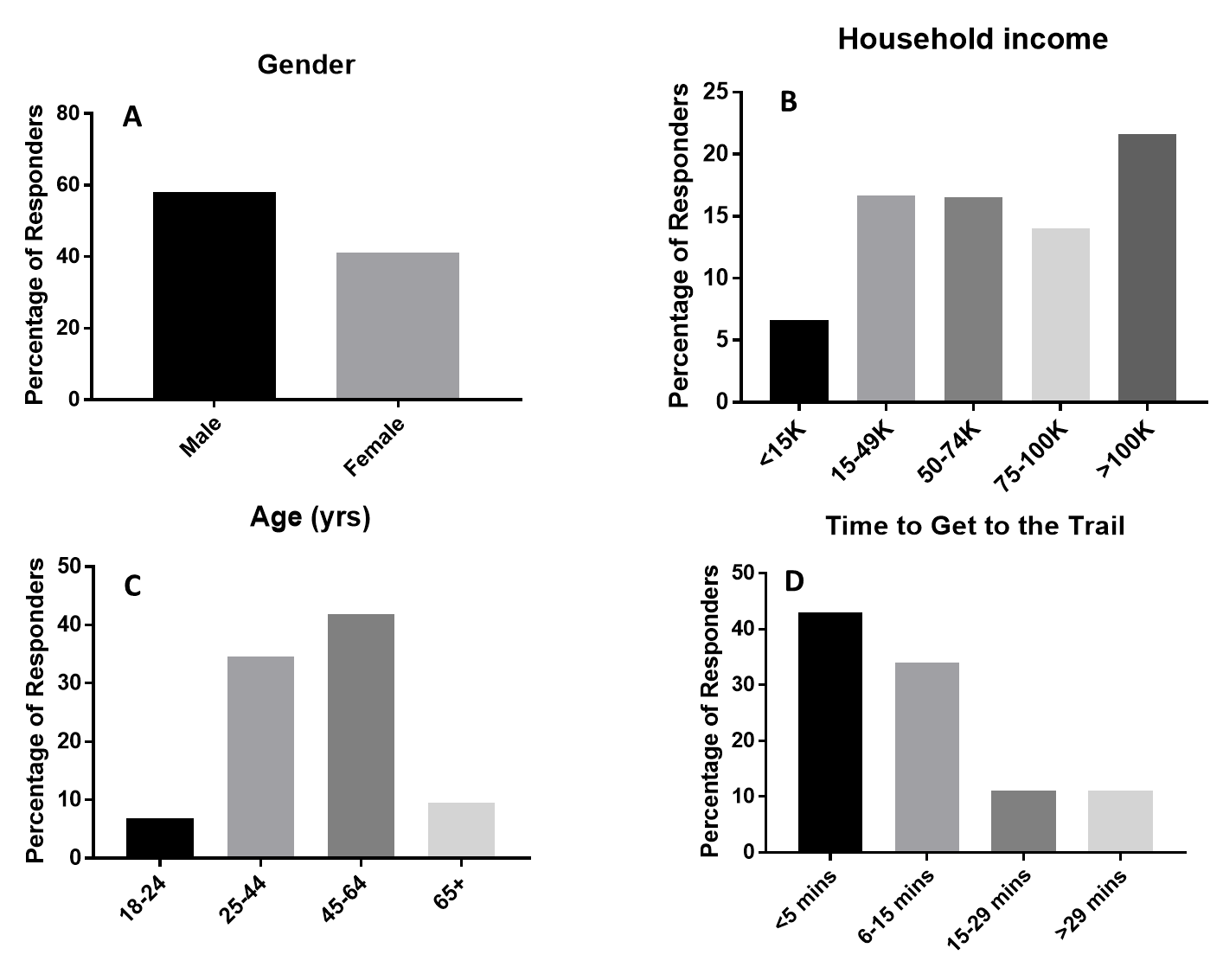


Trail users surveyed while using one of the four recreational multi-use trails were (A) mostly male, (B) over 75% reported a household income over $50,000 CAN; (C) over 50% were aged 45 years or older and (D) over 75% reported that they travelled for 15 minutes or less to use the trail they were surveyed on.

eFigure 4. Parallel trends in CVD events and CVD risk factors prior to the intervention

A


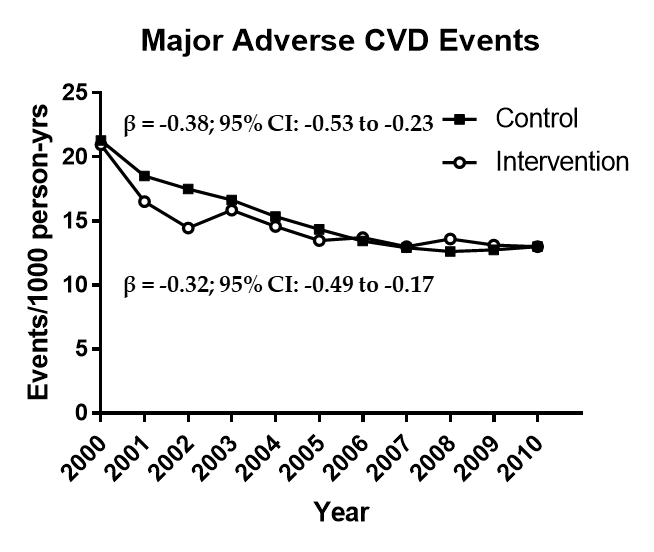


B


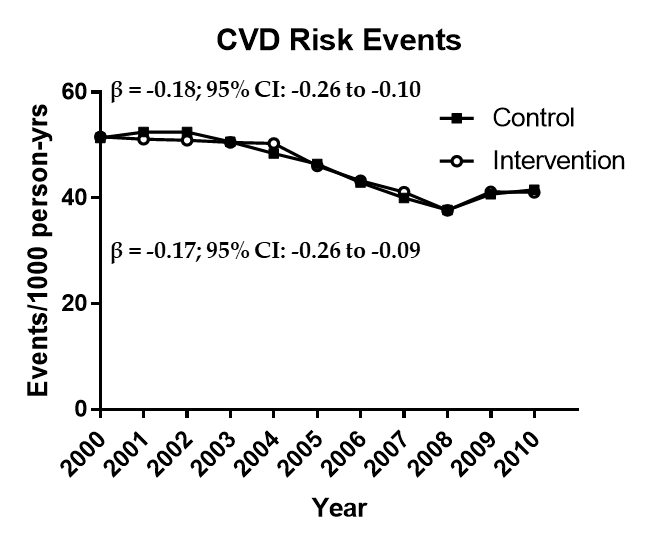


eFigure 5. Sex specific effects of multi-use trails on CVD events and CVD risk factors

A – CVD events


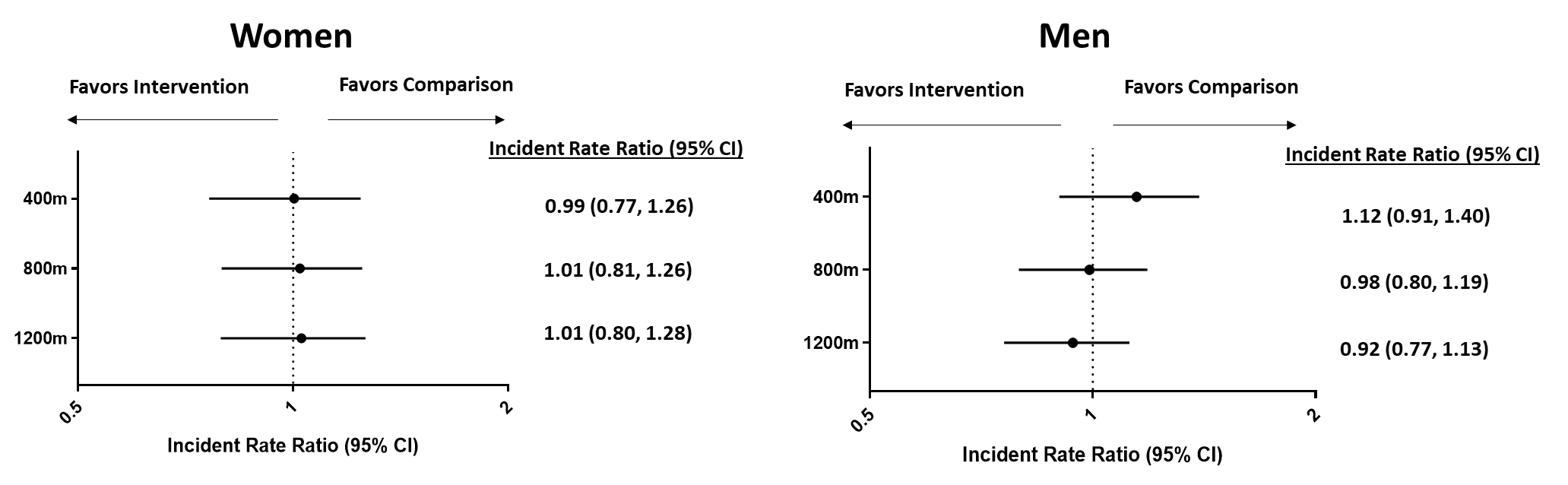


B – CVD risk factors


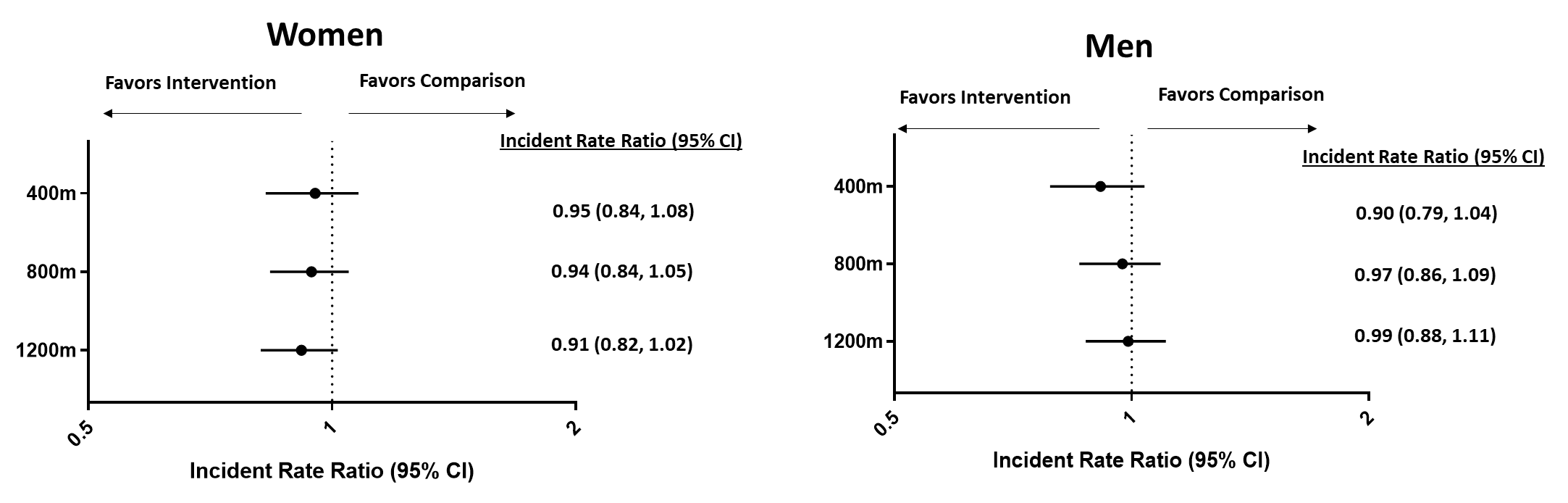


eFigure 6. Effects of multi-use trails in CVD events and CVD risk factors for the population restricted to 30- 65 years


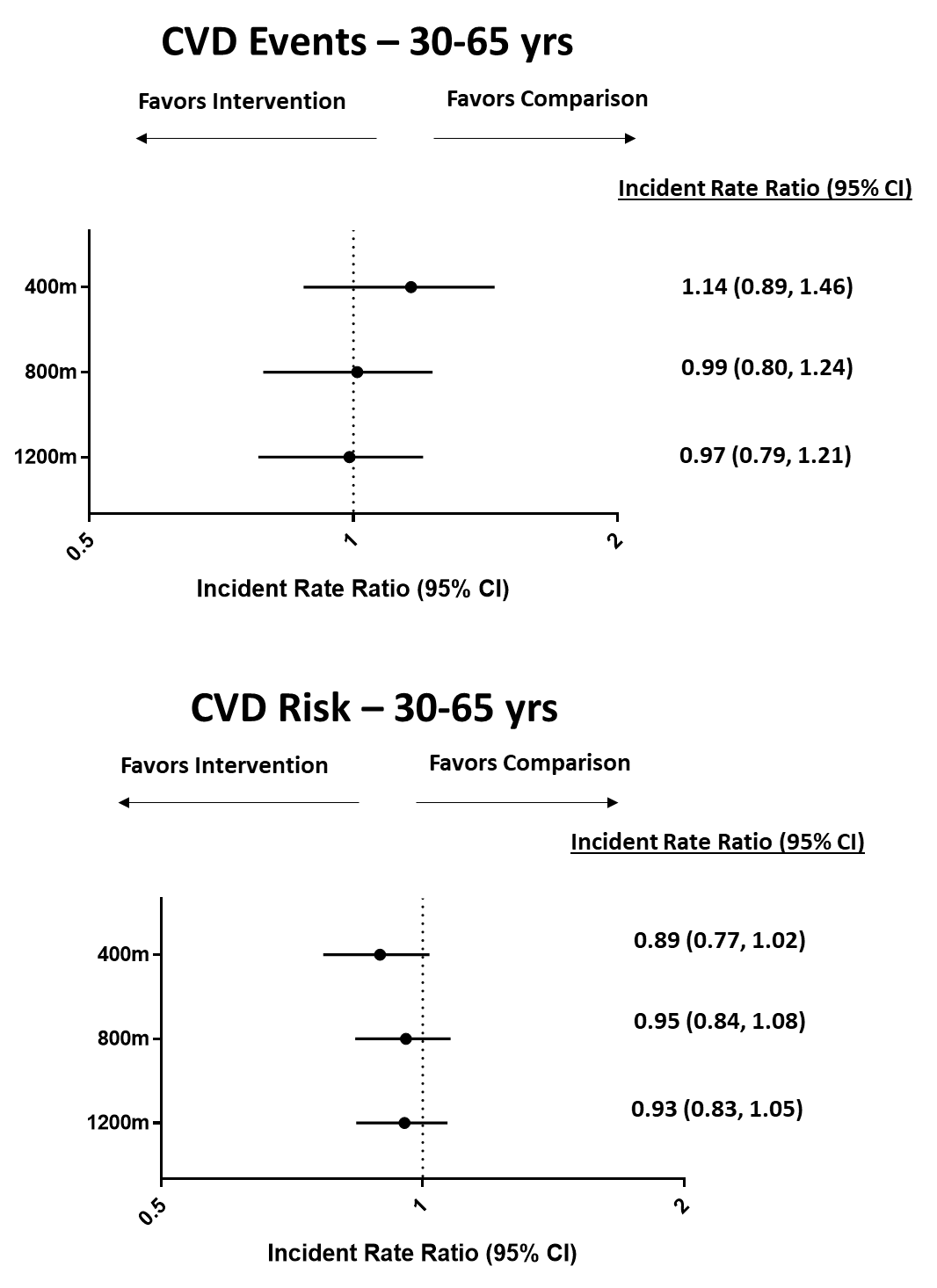


eFigure 7. Effects of multi-use trails on CVD events and CVD risk factors stratified by season

A - CVD Events


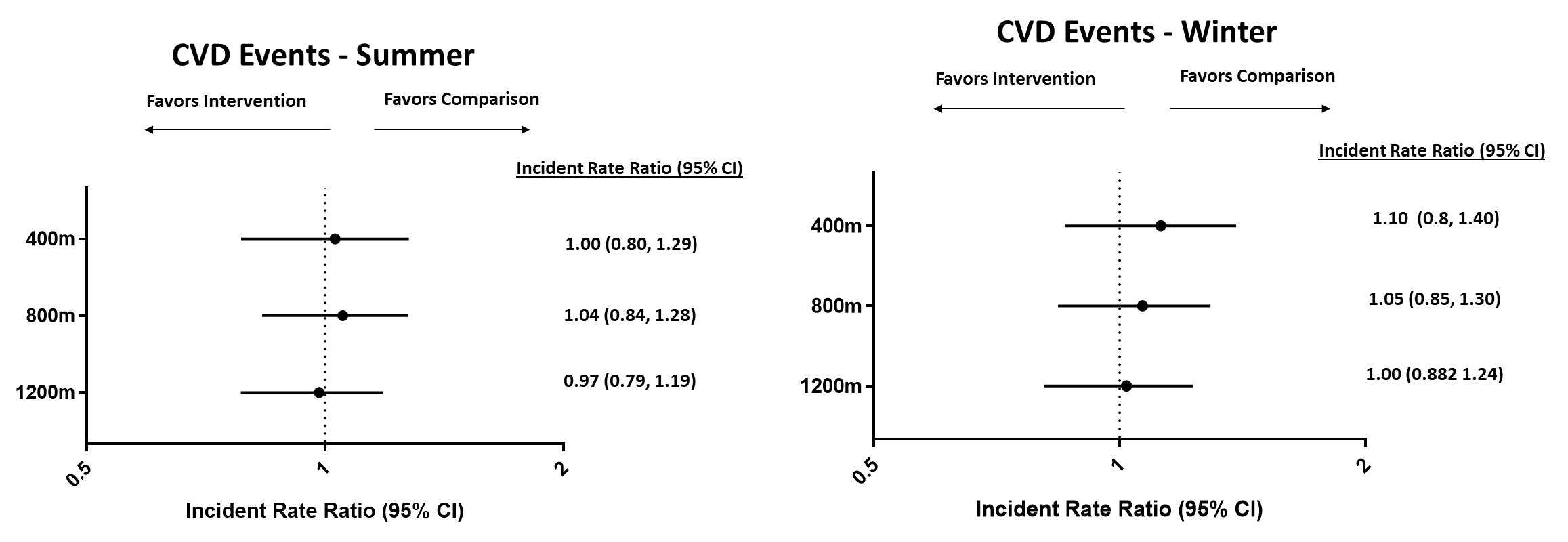


B- CVD Risk Factors


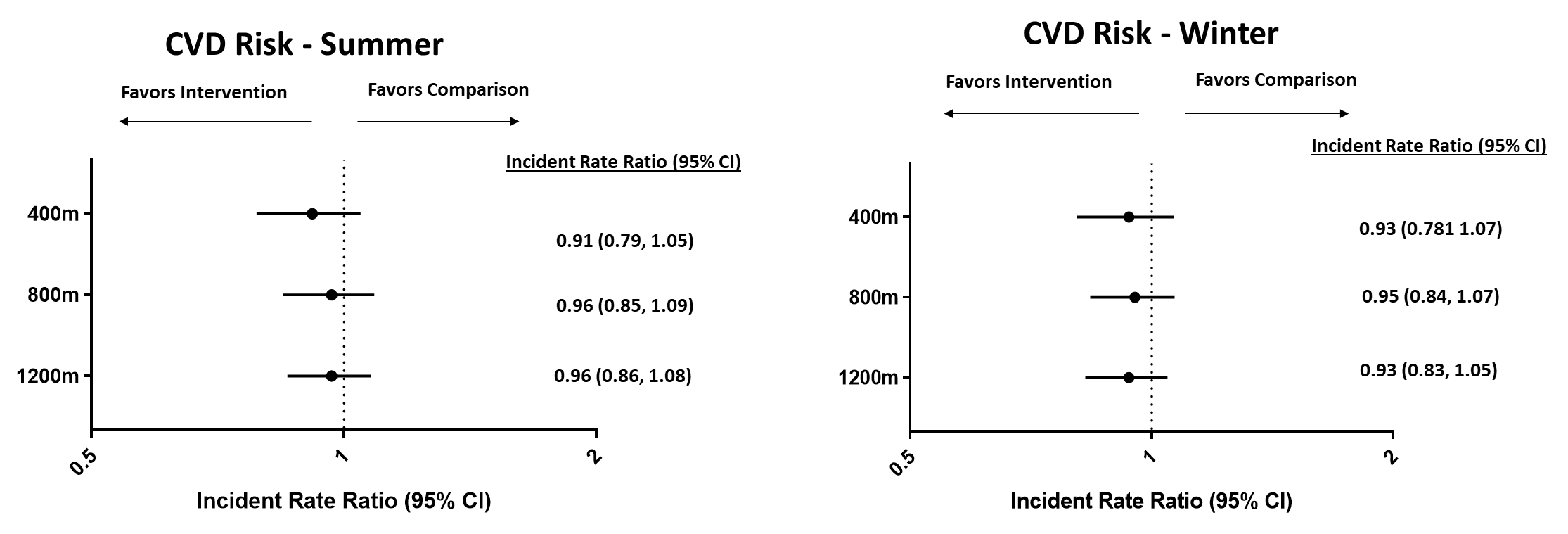


eTable 1. Details of the multi-use trails

| **Variable** | **Trail A** | **Trail B** | **Trail C** | **Trail D** |
| --- | --- | --- | --- | --- |
| Original Land Use | Paved grassland | Converted rail line | Paved grassland | Paved grassland / industrial re-appropriation |
|  |  |  |  |  |
| Const. Start Date | Summer ‘08 | Summer ‘07 | Summer ‘09 | Summer ‘10 |
| Completion date | ~Autumn ‘10 | ~Autumn ‘12 | ~Autumn ‘11 | ~Autumn ‘10 |
| Const. Start Date | Summer ‘08 | Summer ‘07 | Summer ‘09 | Summer ‘10 |
| Completion date | ~Autumn ‘10 | ~Autumn ‘12 | ~Autumn ‘11 | ~Autumn ‘10 |
| Distance of Trail | 8km | 6.5km | 5km | 6.7 km |
|  |  |  |  |  |
| Dissemination areas within 800m access | 60 | 80 | 32 | 35 |
|  |  |  |  |  |
| Estimated pop. within 800m | 26,791 | 31,924 | 12,361 | 12,710 |
|  |  |  |  |  |
| Immediate adjacent environment | Major roadway and neighbourhoods/ business parks | Trail located between two major roadways | Greenspace, neighbourhood, airport | Neighbourhood, grassland, business park |
|  |  |  |  |  |
| Mixed land use | High (homes, schools, shopping, parks) | High (homes, schools, shopping, recreation, parks) | Medium (homes, schools, parks) | Medium (homes, shopping, parks) |

## sTable 2. Cardiovascular disease events and cardiovascular disease risk factor classification and corresponding International Classification of Disease (ICD) codes, in alphabetical order.

| Indication | ICD title | ICD 10 code | ICD 9 code |
| --- | --- | --- | --- |
| **Diagnoses indicative of a cardiovascular disease endpoint – Primary outcome** | | | |
| Ischemic heart disease | Angina pectoris | I20 | 413 |
|  | Acute myocardial infarction | I21 | 410, 434.91 |
|  | Subsequent myocardial infarction | I22 | 412 |
|  | Other acute ischaemic heart diseases | I24 | 411 |
|  | Chronic ischaemic heart disease | I25 | 414 |
| Cardiac arrest | Cardiac arrest | I46 | 427.5 |
| Heart failure | Heart failure | I50 | 428 |
| Cerebral infarction | Cerebral infarction | I63 | 433, 434, 435 |
|  | Stroke | I64 | 437.0, 437.1, 437.3 |
| **Diagnoses indicative of a cardiovascular disease risk factor – Secondary Outcome** | | | |
| Diabetes | Diabetes | E11 | 250 |
| Hyperlipidemia | Dyslipidemia | E78 | 272.3, 272.5, 272.6 |
| Hyperlipidemia | Pure hypercholesterolaemia | E78.0 | 272.0 |
|  | Pure hyperglyceridaemia | E78.1 | 272.1 |
|  | Mixed hyperlipidaemia | E78.2 | 272.2 |
|  | Hyperlipidaemia, unspecified | E78.5 | 272.4 |
| Hypertension | Essential (primary) hypertension | I10 | 401, 459.3 |
|  | Hypertensive heart disease | I11 | 402 |
|  | Hypertensive renal disease | I12 | 403 |
|  | Hypertensive heart and renal disease | I13 | 404 |
| **Diagnoses leading to exclusion from the cohort** | | | |
| Cardiomyopathy | Endocardial fibroelastosis | I42.4 | 425.3 |
|  | Endomyocardial disease | I42.3 | 425.0 |
|  | Congenital cardiomyopathy | I42.8 | Not applicable |
|  | Familial cardiomyopathy | I42.9 | 425.9 |
| Congenital malformation of circulatory system | Congenital malformations of the circulatory system | Q20 to Q28 | 745, 746, 747, 759.9 |
| Cystic fibrosis | Cystic fibrosis | E84 | 277.0 |
| **Ineligible diagnoses (not considered as outcome, but did not warrant exclusion of the participant)** | | | |
| Other cardiovascular problems (eg cause is viral, genetic, alcoholic, etc.) | Other cardiovascular problems | E78.7, E78.8 | 424.9, 425.2, 425.5, 425.7, 425.8, 425.9 |
| Arrhythmia* | Cardiac dysrhythmias | I49 | 427 excluding 427.5 (see Cardiac arrest) |
|  | Abnormalities of heart beat | R00 to 03 | 785.3 |

*Arrhythmias were ineligible because their definition in administrative databases has not yet been validated and a local pediatric cardiologist informed us that most dysrhythmia diagnoses or referrals would not be considered cardiovascular disease or risk.

sTable 3. Definitions and sources of data to define cardiovascular disease events and cardiovascular disease risk factors

| **Category** | **Variable** | **Outcome** | **Definition** | **Source** |
| --- | --- | --- | --- | --- |
| Primary | Major adverse cardiovascular events (MACE) - Composite | CVD-related mortality | Death in vital statistics mortality data with most responsible cause of death coded as CHF, IHD or stroke. | Vital statistics mortality |
|  |  | Ischemic heart disease | -1+ Inpatient Hospitalizations  -2+ Physician visits in 5 years  -1 Physician Visit & 2+ Rx in 5 years | Hospital abstracts, Medical claims, DPIN prescription dispensations |
|  |  |  |  |  |
|  |  | Congestive heart failure | -1+ inpatient visits or 2+ physician visits | Hospital abstracts, Medical claims |
|  |  |  |  |  |
|  |  | Cerebrovascular event | - 1+ Inpatient Hospitalizations  - death in hospital | Hospital abstracts |
|  |  |  |  |  |
| Secondary | CVD-related risk factors - Composite | Hypertension | -1+ Inpatient Hospitalizations  -2+ Physician visits in 2 years | Hospital abstracts,  Medical claims |
|  |  | Diabetes | -1+ Inpatient Hospitalizations  -2+ Physician visits in 3 years  -2+ Rx for glucose lowering agents in 3 years, | Hospital abstracts,  Medical claims, Prescription dispensations |
|  |  | Dyslipidemia | -1+ Hospitalization  -2+ Physician visits in 3 years  -2+ Rx for statins in 3 years | Hospital abstracts,  Medical claims, Prescription dispensations |
|  |  |  |  |  |
| Secondary | Trail use | Bicycle counts |  | Eco-Counter Magnetic Zelts located beneath greenways |
|  |  |  |  |  |
| Exploratory | Income | Household | Self-reported | Intercept surveys |
|  | Ethnicity |  | Self-reported |  |
|  | Gender | Male, female, other | Self-reported |  |
|  | Residence | Postal code | Self-reported |  |
|  | Weekly use | Visits/week | Self-reported |  |
|  | Time on trail | Minutes/use | Self-reported |  |

CHF = congestive heart failure; CVD = cardiovascular disease; ICD = international classification code; IHD = ischemic heart disease, Rx = prescription.

eTable 4. Area-level descriptive variables for each multi-use trail.

|  | **Trail A** | **Trail B** | **Trail C** | **Trail D** |
| --- | --- | --- | --- | --- |
| Dissemination Areas* (DA) | 60 | 80 | 32 | 35 |
| Female** (%) | 53.26% | 53.12% | 52.80% | 51.43% |
| Age** (years) | 53.00 | 54.00 | 52.95 | 50.81 |
| Visible Minority* (%) | 15.60% | 10.82% | 6.88% | 6.37% |
| Immigrated last 10 years* (%) | 4.95% | 3.97% | 2.47% | 1.80% |
| ***Socioeconomic indicators*** |  |  |  |  |
| SEFI** | -0.6240 | -0.2165 | -0.3670 | -0.3108 |
| Household income* | $78,903.78 | $67,918.18 | $64,027.56 | $68,255.49 |
| Average Property Value† | $150,949.41 | $116,183.20 | $104,129.83 | $106,676.21 |
| Population without high school graduation* (%) | 19.57% | 25.39% | 19.30% | 24.40% |
| Unemployment Rate* | 5.15% | 4.79% | 4.22% | 4.97% |
| **Physical Activity indicators** |  |  |  |  |
| Fitness/Recreation Centres within 5km† (2018 only) | 96.20 | 100% | 61.82% | 100% |
| Average Distance to greenspace† (m) | 192.65 | 154.89 | 162.92 | 160.99 |
| Walkability Score‡ | -0.0798 | 0.4680 | 0.0726 | -0.0366 |
| Active commuting* (%) | 6.25% | 5.03% | 5.59% | 3.47% |
